# Exceptionally Durable Response to First-Line S-1 Monotherapy in Metastatic Pancreatic Adenocarcinoma: A Case Report

**DOI:** 10.7759/cureus.116149

**Published:** 2026-09-12

**Authors:** Takayuki Ota, Shoji Iinuma, Yosuke Suyama

**Affiliations:** 1 Gastroenterology, Rakuwakai Marutamachi Hospital, Kyoto, JPN; 2 Gastroenterology, Rakuwakai Otowa Hospital, Kyoto, JPN

**Keywords:** chemotherapy, exceptional response, liver metastasis, pancreatic adenocarcinoma, s-1

## Abstract

Pancreatic adenocarcinoma with distant metastases is associated with a poor prognosis, and exceptionally durable responses to systemic chemotherapy are uncommon. We report a rare case of pancreatic adenocarcinoma with radiologically suspected multiple liver metastases that achieved a prolonged response to first-line tegafur/gimeracil/oteracil (S-1) monotherapy.

A 76-year-old man with a history of cerebral infarction and diabetes mellitus was referred to our department because of rapidly worsening diabetes. Imaging revealed an approximately 3-cm pancreatic head tumor and multiple hepatic lesions highly suspicious for metastases. Serum carbohydrate antigen 19-9 (CA19-9) was markedly elevated. Endoscopic ultrasound-guided fine-needle aspiration (EUS-FNA) of the pancreatic head tumor demonstrated adenocarcinoma on both cytological and histopathological examinations. Gemcitabine plus nanoparticle albumin-bound paclitaxel (nab-paclitaxel) was proposed; however, the patient preferred oral treatment. Considering his age, quality of life, and treatment preference, S-1 monotherapy was initiated at 100 mg/day on a 2-week-on/2-week-off schedule.

Following initiation of S-1, the pancreatic and hepatic lesions progressively regressed, accompanied by rapid normalization of CA19-9. By approximately 14 months after treatment initiation, the previously identifiable pancreatic primary tumor and hepatic lesions were no longer clearly visualized on computed tomography (CT). S-1 monotherapy was continued with few interruptions, and no apparent radiological progression was documented for approximately 47 months.

Approximately two and a half months after the last CT showing no apparent progression, the patient developed fever and dyspnea. CT demonstrated newly developed bilateral ground-glass and interstitial pulmonary opacities, along with new-onset ascites, without evidence of regrowth of the pancreatic or hepatic lesions. S-1-induced interstitial lung disease (ILD) was clinically suspected; however, occult malignant progression could not be definitively excluded. His respiratory condition deteriorated despite treatment, and he subsequently died.

This case demonstrates that an exceptionally durable response may rarely occur with first-line S-1 monotherapy even in pancreatic adenocarcinoma with radiologically suspected metastatic disease.

## Introduction

Pancreatic cancer is an aggressive malignancy that is frequently diagnosed at an advanced stage. Patients with metastatic disease have particularly poor outcomes, and systemic chemotherapy remains the mainstay of treatment. Combination regimens, such as 5-fluorouracil, leucovorin, irinotecan, and oxaliplatin (FOLFIRINOX) and gemcitabine plus nanoparticle albumin-bound paclitaxel (nab-paclitaxel), have improved outcomes in patients with metastatic pancreatic cancer; however, long-term disease control remains uncommon [1,2]. More recently, updated guidelines have incorporated NALIRIFOX among first-line treatment options for metastatic pancreatic cancer, alongside established combination regimens such as FOLFIRINOX and gemcitabine plus nab-paclitaxel.

Tegafur/gimeracil/oteracil (S-1) is an oral fluoropyrimidine formulation. In a randomized phase III study, S-1 monotherapy demonstrated noninferiority to gemcitabine with respect to overall survival in patients with locally advanced or metastatic pancreatic cancer, establishing its antitumor activity in advanced pancreatic cancer [3]. Nevertheless, exceptionally durable responses to S-1 monotherapy are uncommon. S-1 consists of tegafur, a prodrug of 5-fluorouracil (5-FU), combined with gimeracil, which inhibits 5-FU degradation, and oteracil, which reduces gastrointestinal toxicity. Although S-1 monotherapy has established activity in advanced pancreatic cancer, it is generally used selectively according to patient factors, such as age, performance status, tolerability, and treatment preference, rather than as a preferred regimen for all patients with metastatic disease [3].

Several case reports have described marked or complete responses to S-1 in pancreatic cancer [4-9]. However, reports documenting the prolonged disappearance of radiologically identifiable disease following first-line S-1 monotherapy alone in previously untreated pancreatic adenocarcinoma with synchronous multiple liver metastases are extremely limited.

Here, we report a patient with histologically confirmed pancreatic adenocarcinoma and radiologically suspected synchronous multiple liver metastases who achieved an exceptionally durable response following first-line S-1 monotherapy without surgery, radiotherapy, or any other systemic anticancer therapy.

## Case presentation

A 76-year-old man with a history of cerebral infarction and diabetes mellitus was referred to our department because of rapidly worsening diabetes mellitus. His height was 160 cm and body weight was 60 kg, corresponding to a body mass index of 23.4 kg/m² and a body surface area of 1.63 m². Abdominal ultrasonography revealed a 24 × 20-mm mass in the pancreatic head and a 24 × 15-mm hepatic lesion in segment 3.

Contrast-enhanced computed tomography (CT) performed on May 23, 2022, demonstrated an irregular 30 × 39-mm mass in the pancreatic head, accompanied by upstream main pancreatic duct dilatation and pancreatic parenchymal atrophy. A hypoenhancing hepatic lesion was identified in the lateral segment of the liver. Magnetic resonance imaging (MRI) performed on May 30, 2022, demonstrated an approximately 3-cm pancreatic head mass with diffusion restriction and distal main pancreatic duct dilatation. Diffusion-weighted imaging (DWI) revealed a total of 6 hyperintense hepatic lesions involving both hepatic lobes, with the largest lesion measuring 25 × 15 mm in the lateral segment. In the clinical context of pancreatic malignancy, their multiplicity, bilobar distribution, hypoenhancement on CT, and diffusion abnormalities were considered highly suspicious for synchronous hepatic metastases. Regional lymph node metastasis was also suspected (Figure 1).

**Figure 1:**
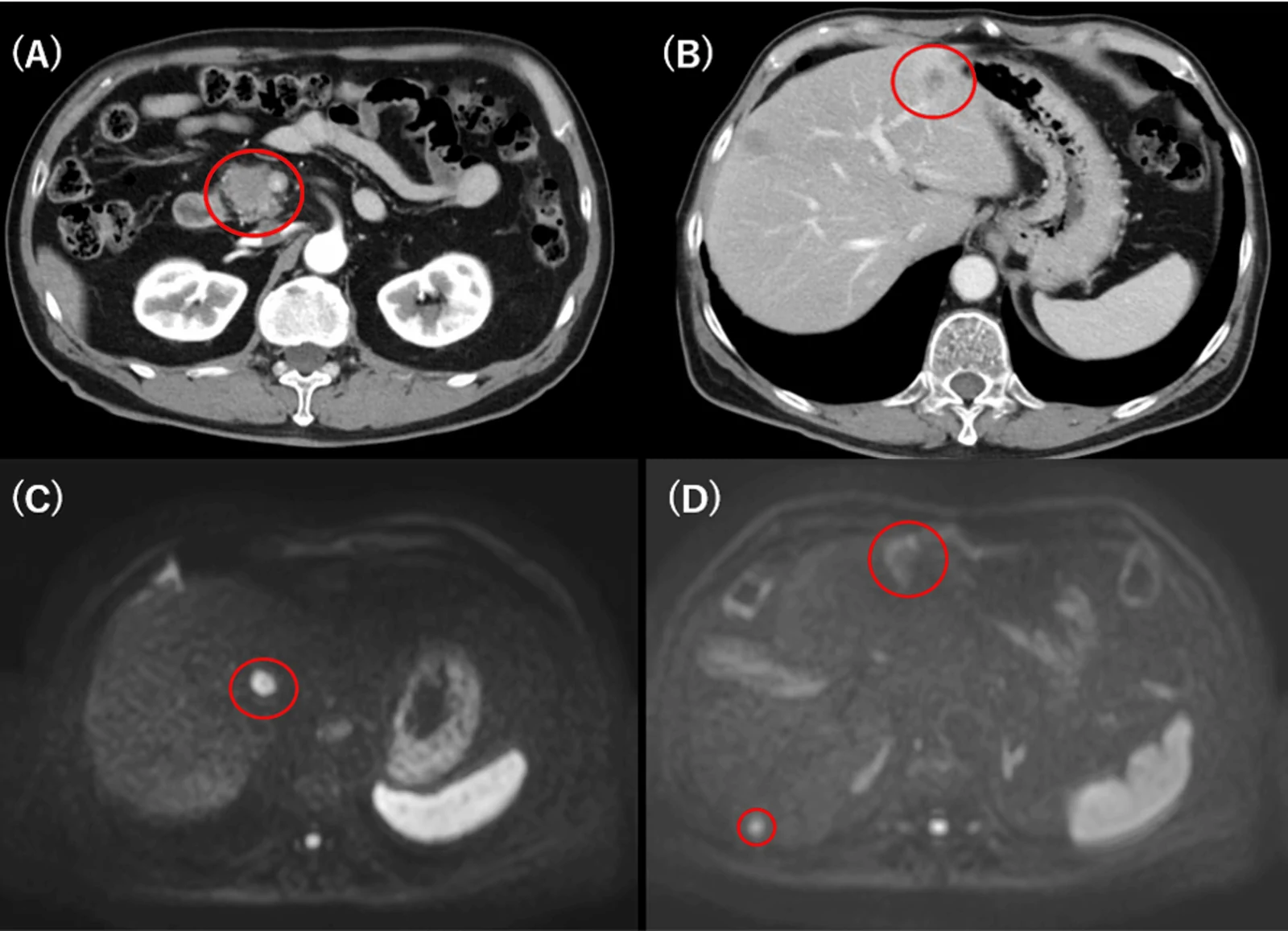
Initial imaging findings Contrast-enhanced computed tomography (CT) obtained on May 23, 2022, demonstrates an irregular 30 × 39-mm pancreatic head tumor (A, red circle) and a representative hypoenhancing lesion in the lateral segment of the liver suspicious for metastasis (B, red circle). Diffusion-weighted magnetic resonance imaging (MRI) obtained on May 30, 2022, demonstrates multiple hyperintense hepatic lesions involving both hepatic lobes (C, D, red circles). A total of 6 hepatic lesions were identified, with the largest lesion measuring 25 × 15 mm in the lateral segment, findings highly suspicious for multiple liver metastases.

The serum carbohydrate antigen 19-9 (CA19-9) level was markedly elevated at 3,750 U/mL (reference range, ≤37 U/mL) at presentation and subsequently increased to a maximum of 5,930 U/mL. The serum carcinoembryonic antigen (CEA) level at presentation was 2.8 ng/mL (reference range, ≤5.0 ng/mL). Baseline laboratory findings additionally demonstrated poorly controlled diabetes, with a serum glucose level of 343 mg/dL (reference range, 70-110 mg/dL) and hemoglobin A1c level of 10.8% (reference range, 4.6%-6.2%).

EUS-FNA of the pancreatic head tumor was subsequently performed. Cytological examination was positive for malignancy and demonstrated adenocarcinoma. Histopathological examination also demonstrated adenocarcinoma, with atypical cells showing nuclear irregularity and anisokaryosis forming small nests and infiltrating the stroma. These findings established the diagnosis of pancreatic adenocarcinoma.

Histological confirmation of the hepatic lesions was not pursued because adenocarcinoma had already been established by EUS-FNA of the pancreatic primary tumor, the multiple bilobar hepatic lesions were radiologically highly suspicious for metastases, and additional liver biopsy was not expected to alter the treatment strategy. Nevertheless, because the hepatic lesions were not biopsied, alternative diagnoses could not be completely excluded. The disease was therefore clinically classified as stage IV pancreatic adenocarcinoma based on the radiologically suspected hepatic metastases.

His Eastern Cooperative Oncology Group (ECOG) performance status was 0. His regular medications included sitagliptin 50 mg/day, glimepiride 1 mg/day, and dapagliflozin 10 mg/day for diabetes mellitus, as well as aspirin 100 mg/day, rabeprazole 10 mg/day, and atorvastatin 5 mg/day.

Gemcitabine plus nab-paclitaxel combination chemotherapy was proposed. After discussion of the expected efficacy, adverse event profiles, route of administration, and treatment burden of available systemic therapies, the patient expressed a strong preference for oral treatment and wished to avoid intravenous combination chemotherapy. Following shared decision-making, first-line S-1 monotherapy was selected.

S-1 was initiated on June 16, 2022, at a dose of 100 mg/day using a 2-week-on/2-week-off schedule. Based on his body surface area of 1.63 m², the standard initial S-1 dose was 120 mg/day; therefore, 100 mg/day represented a 1-step dose reduction. This reduction was selected in consideration of his age and the potential risk of treatment-related adverse events rather than renal impairment. At treatment initiation, his serum creatinine level was 0.50 mg/dL and estimated glomerular filtration rate was 118 mL/min/1.73 m², indicating preserved renal function. Treatment was generally continued according to the planned schedule. In April and June 2026, one treatment cycle was skipped on each occasion because of fever, resulting in two treatment interruptions. S-1 was subsequently resumed after recovery. No further dose reduction was required.

Following initiation of S-1, the pancreatic primary tumor and hepatic lesions progressively decreased in size. The radiological response was accompanied by a marked decrease in CA19-9, which fell from a maximum of 5,930 U/mL to 235 U/mL approximately 2 months after treatment initiation and normalized to 33.6 U/mL approximately 3 months after treatment initiation.

In a retrospective assessment according to Response Evaluation Criteria in Solid Tumors (RECIST) version 1.1, the pancreatic head tumor and the largest hepatic lesion were selected as measurable target lesions. At baseline, their longest diameters were 39 mm and 25 mm, respectively, giving a sum of 64 mm. On July 20, 2022, the pancreatic and hepatic lesions measured 23 mm and 20 mm, respectively, giving a sum of 43 mm. This represented a 32.8% decrease from baseline and met the criteria for partial response (PR). Thus, the time to the first objectively documented response was 34 days after initiation of S-1. The previously suspected regional lymphadenopathy was also indistinct on this examination.

On October 26, 2022, the pancreatic lesion measured 16 mm, while the hepatic lesions and regional lymphadenopathy were no longer clearly visualized. On March 29, 2023, the pancreatic lesion measured approximately 10 mm, with the hepatic lesions and lymphadenopathy remaining indistinct. On August 16, 2023, the previously identified pancreatic and hepatic lesions were no longer clearly visualized on contrast-enhanced CT (Figure 2). Because retrospective assessment of routine clinical imaging did not permit unequivocal confirmation of all RECIST 1.1 criteria for complete response, the best objectively documented response was conservatively classified as PR.

**Figure 2:**
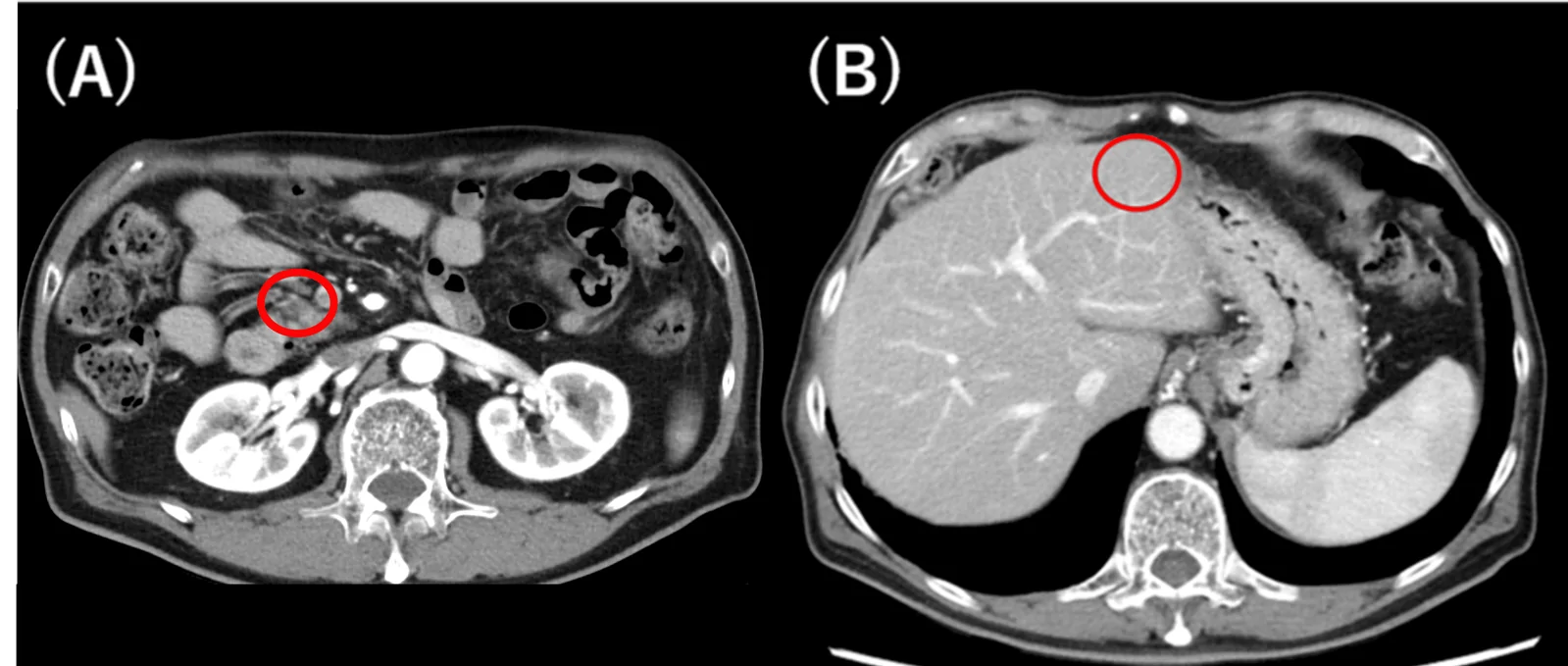
Radiological response to S-1 monotherapy Contrast-enhanced computed tomography obtained on August 16, 2023, approximately 14 months after initiation of S-1 monotherapy, demonstrates that the previously identified pancreatic head tumor (A) and hepatic lesion in the lateral segment (B) are no longer clearly visualized. The images were selected at anatomical levels corresponding as closely as possible to those shown on baseline CT. No new metastatic lesions were identified.

S-1 monotherapy was continued without additional systemic chemotherapy, radiotherapy, or surgical resection. CA19-9 remained within the normal range throughout subsequent follow-up. Serial CT examinations demonstrated no definite radiological progression through May 8, 2026. The documented response was therefore maintained for at least approximately 46 months from the first PR, and the documented progression-free interval from initiation of S-1 was approximately 47 months. His ECOG performance status remained 0 during most of the treatment course but declined to 1 around June 2026.

On July 29, 2026, the patient was admitted because of fever and progressive dyspnea. His ECOG performance status was 1. CT demonstrated newly developed bilateral ground-glass and interstitial pulmonary opacities, along with new-onset ascites, without evidence of regrowth of the previously identified pancreatic primary tumor or hepatic lesions (Figure 3).

**Figure 3:**
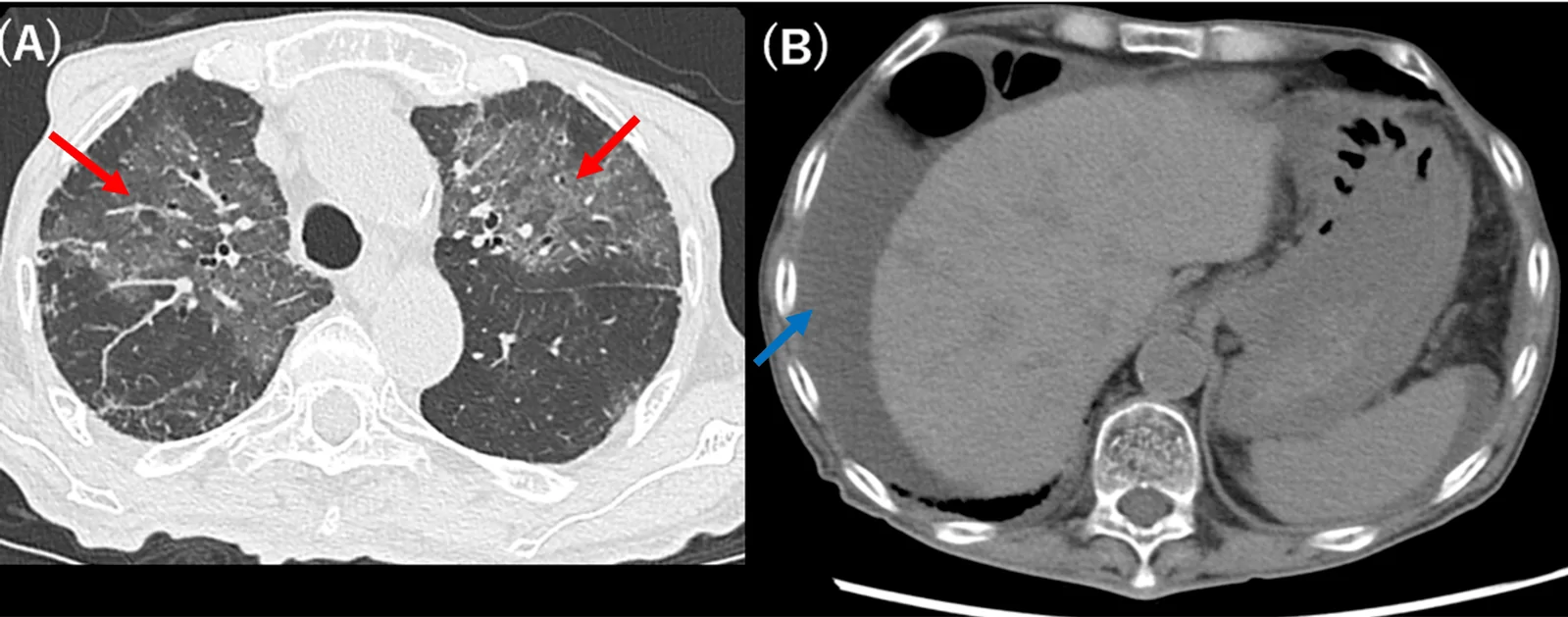
Computed tomography findings during the terminal clinical event Computed tomography obtained on July 29, 2026, demonstrates newly developed bilateral ground-glass and interstitial pulmonary opacities on lung-window imaging (A, red arrows) and new-onset ascites on abdominal CT (B, blue arrow). No obvious regrowth of the previously identified pancreatic primary tumor or hepatic lesions was identified. The etiology of the pulmonary abnormalities and ascites remained uncertain.

At admission, the white blood cell count was 5.8 × 10³/μL (reference range, 4.0-8.0 × 10³/μL), C-reactive protein was 10.44 mg/dL (reference range, 0-0.24 mg/dL), lactate dehydrogenase was 426 U/L (reference range, 124-222 U/L), and serum albumin was 2.6 g/dL (reference range, 4.1-5.1 g/dL). Krebs von den Lungen-6 was 495 U/mL (reference range, 0-500 U/mL). Testing for coronavirus disease 2019 and influenza was negative, and serological testing for autoimmune diseases was unrevealing. CA19-9 remained within the normal range at 10.9 U/mL (reference range, 0-37 U/mL), whereas CEA was mildly elevated at 8.6 ng/mL (reference range, 0-5.0 ng/mL). No dedicated cardiac evaluation was performed during the terminal admission.

Empirical ceftriaxone at 2 g/day was administered from admission for possible pulmonary infection. Oral corticosteroid therapy at 50 mg/day was initiated on July 30 for suspected drug-induced interstitial lung disease. Because his respiratory condition worsened, intravenous methylprednisolone pulse therapy at 1,000 mg/day for 3 days was initiated on July 31, followed by continued oral corticosteroid therapy. Despite treatment, his respiratory condition progressively deteriorated, and he died on August 7, 2026. Overall survival from initiation of S-1 was approximately 50 months.

No lung biopsy or cytological examination of the ascites was performed. Therefore, the etiology of the terminal pulmonary abnormalities and ascites could not be definitively established. Drug-induced interstitial lung disease, pulmonary infection, pulmonary lymphangitic carcinomatosis, and peritoneal progression remained competing explanations. Accordingly, although no definite radiological progression had been documented through May 8, 2026, the terminal event could not be definitively classified as either malignant progression or a nonmalignant pulmonary process.

A CARE-compliant timeline summarizing the diagnostic evaluation, S-1 treatment, CA19-9 changes, radiological response, and terminal clinical course is provided in Table 1.

**Table 1:** Clinical timeline of diagnosis, S-1 treatment, radiological response, and terminal clinical course DWI, diffusion-weighted imaging; ECOG PS, Eastern Cooperative Oncology Group performance status; EUS-FNA, endoscopic ultrasound-guided fine-needle aspiration; MRI, magnetic resonance imaging; PR, partial response; RECIST, Response Evaluation Criteria in Solid Tumors; S-1, tegafur/gimeracil/oteracil

Date	Clinical event/treatment	Imaging/response	CA19-9
May 11, 2022	Initial evaluation for worsening diabetes; ECOG PS 0	—	3,750 U/mL around presentation
May 23, 2022	Diagnostic evaluation	Contrast-enhanced CT: pancreatic head tumor 30 × 39 mm; hypoenhancing hepatic lesion	—
May 30, 2022	Staging MRI	Approximately 3-cm pancreatic tumor; six DWI-hyperintense hepatic lesions involving both lobes; largest 25 × 15 mm; regional lymph-node metastasis suspected	—
May-June 2022	EUS-FNA confirmed pancreatic adenocarcinoma; liver lesions not biopsied; clinical stage IV based on radiologically suspected hepatic metastases	—	Maximum 5,930 U/mL
June 16, 2022	S-1 initiated at 100 mg/day, 2 weeks on/2 weeks off	Baseline retrospective RECIST target-lesion sum: 64 mm	—
July 20, 2022	S-1 continued	Pancreas 23 mm + liver 20 mm = 43 mm; 32.8% decrease; PR by retrospective RECIST 1.1; regional lymphadenopathy indistinct	—
~August 2022	S-1 continued	—	235 U/mL
~September 2022	S-1 continued	—	33.6 U/mL
October 26, 2022	S-1 continued	Pancreatic lesion 16 mm; hepatic lesions and lymphadenopathy no longer clearly visualized	Normal range
March 29, 2023	S-1 continued	Pancreatic lesion approximately 10 mm; hepatic lesions and lymphadenopathy remained indistinct	Normal range
August 16, 2023	S-1 continued	Pancreatic and hepatic lesions no longer clearly visualized; no new metastatic lesions	Normal range
April 2026	Fever; one S-1 treatment cycle skipped	—	—
May 8, 2026	S-1 continued	Last CT documenting no definite radiological progression	Normal range
June 2026	Fever; one S-1 treatment cycle skipped; ECOG PS declined to 1	—	—
July 29, 2026	Hospitalized with fever and dyspnea; ECOG PS 1; ceftriaxone 2 g/day initiated	Bilateral ground-glass/interstitial pulmonary opacities and new ascites; no obvious pancreatic or hepatic regrowth	10.9 U/mL
July 30, 2026	Oral corticosteroid therapy 50 mg/day initiated	—	—
July 31-August 2, 2026	Methylprednisolone 1,000 mg/day for 3 days, followed by oral corticosteroid therapy 50 mg/day	Progressive respiratory deterioration	—
August 7, 2026	Death	Terminal etiology remained unresolved	—

## Discussion

The present case is notable for an exceptionally profound and durable response to first-line S-1 monotherapy in a patient with pancreatic adenocarcinoma and radiologically suspected metastatic disease. At diagnosis, the patient had histologically confirmed pancreatic adenocarcinoma, radiologically suspected synchronous multiple liver metastases, and markedly elevated CA19-9. Following initiation of S-1 monotherapy, both the pancreatic primary tumor and hepatic lesions progressively regressed, accompanied by rapid normalization of CA19-9. In a retrospective RECIST 1.1 assessment, a PR was documented 34 days after initiation of S-1. By approximately 14 months after treatment initiation, the previously identifiable pancreatic and hepatic lesions were no longer clearly visualized on contrast-enhanced CT. No definite radiological progression was documented through May 8, 2026, approximately 47 months after treatment initiation.

The duration of disease control in the present case is particularly notable in the context of outcomes generally observed in metastatic pancreatic cancer. In the pivotal phase III trial of FOLFIRINOX, median overall survival was 11.1 months, and median progression-free survival was 6.4 months [1]. Similarly, in the phase III MPACT trial, gemcitabine plus nab-paclitaxel resulted in a median overall survival of 8.5 months and median progression-free survival of 5.5 months [2]. Although direct comparison between an individual case and clinical trial populations is inappropriate, the prolonged absence of definite radiological progression observed in our patient is clearly unusual.

S-1 has established activity against advanced pancreatic cancer. In the randomized phase III GEST study, S-1 monotherapy was noninferior to gemcitabine in terms of overall survival [3]. However, exceptionally durable responses to S-1 monotherapy appear to be rare.

Several previously published cases provide useful comparisons. Mizuno et al. reported a patient with advanced pancreatic cancer treated with S-1 monotherapy in whom two liver metastases disappeared completely; however, the overall response remained a partial response [4]. Yoshino et al. described a chemotherapy-naïve patient with advanced pancreatic cancer and multiple liver metastases who achieved a marked response to S-1 monotherapy, but the overall response was also classified as a partial response [5].

Sato et al. reported an elderly patient with stage II pancreatic cancer who achieved a complete response following low-dose S-1 monotherapy [6]. Unlike the present patient, however, distant metastatic disease was absent. Saso et al. reported a patient who developed multiple liver metastases following pancreaticoduodenectomy and subsequently achieved a complete response to S-1 monotherapy [7]. However, this represented postoperative recurrence, and the primary tumor showed unusual histological features.

Miyata et al. described a patient with pancreatic head cancer and liver metastasis who ultimately achieved a pathological complete response [8]. That patient received multimodal treatment, including S-1-based chemoradiotherapy and subsequent surgery, making the treatment course substantially different from S-1 monotherapy alone. Yamaga et al. reported a long-term complete response to S-1 monotherapy for para-aortic lymph node recurrence of pancreatic ductal adenocarcinoma [9]. In that case, however, the patient had previously undergone surgical resection and adjuvant gemcitabine therapy.

As summarized in Table 2, previously reported cases were heterogeneous with respect to disease setting, method of metastatic confirmation, treatment exposure, response assessment, response duration, and outcome. These differences limit direct comparison with the present case. The present case differs from these reports in several important respects. S-1 was administered as first-line monotherapy for previously untreated pancreatic adenocarcinoma with synchronous radiologically suspected multiple liver metastases. No surgery, radiotherapy, or additional systemic anticancer agent was administered. A PR according to a retrospective RECIST 1.1 assessment was documented 34 days after initiation of S-1 and was maintained for at least approximately 46 months. Although the lesions subsequently became no longer clearly visualized, the best objectively documented response was conservatively classified as PR because all criteria required for a formal complete response could not be unequivocally confirmed retrospectively.

**Table 2:** Selected reports of marked or complete responses to S-1-containing treatment in pancreatic cancer CR, complete response; PR, partial response; PDAC, pancreatic ductal adenocarcinoma; RECIST, Response Evaluation Criteria in Solid Tumors; S-1, tegafur/gimeracil/oteracil

Report	Disease setting	Metastatic confirmation	S-1 treatment	Response assessment	Best response	Response duration/outcome
Mizuno et al. [4]	Inoperable advanced pancreatic cancer with liver metastases	Radiological	S-1 monotherapy	Imaging-based assessment after 4 courses	Liver lesions: CR; overall: PR	Marked response after 4 courses
Yoshino et al. [5]	Chemotherapy-naïve advanced pancreatic cancer with multiple liver metastases	Radiological	First-line S-1 monotherapy	RECIST	PR	PR documented after 4 courses
Sato et al. [6]	Stage II localized pancreatic cancer in an elderly patient	No distant metastasis	Low-dose S-1 monotherapy, 50 mg/day, 28 days on/14 days off	CT-based	CR	CR after 5 courses
Saso et al. [7]	Postoperative multiple liver recurrence	Radiological	S-1 monotherapy, 80 mg/day, 4 weeks on/2 weeks off	Contrast-enhanced CT	PR → CR	PR at 3 months; CR at 9 months; CR maintained >12 months
Miyata et al. [8]	Pancreatic head cancer with liver metastasis	Pathologically confirmed liver metastasis	S-1-containing multimodal therapy including chemoradiotherapy, S-1, and surgery	Imaging and pathological assessment	Pathological CR	Alive without recurrence 24 months after surgery
Yamaga et al. [9]	Postoperative para-aortic lymph-node recurrence of PDAC	Radiological	S-1 monotherapy after surgery and adjuvant gemcitabine	Imaging-based	CR	Long-term CR
Present case	Untreated pancreatic adenocarcinoma with synchronous multiple hepatic lesions	Radiologically suspected; no liver biopsy	First-line S-1 monotherapy, 100 mg/day, 2 weeks on/2 weeks off	Retrospective RECIST 1.1	PR; lesions subsequently no longer clearly visualized	PR at day 34; response maintained ≥46 months; no definite progression through May 8, 2026

The marked decline in CA19-9 provides additional support for the profound treatment response. CA19-9 reached a maximum of 5,930 U/mL around treatment initiation but decreased to 235 U/mL approximately 2 months after initiation of S-1 and normalized to 33.6 U/mL approximately one month later. It subsequently remained within the normal range throughout long-term follow-up. The parallel regression of the radiological abnormalities and normalization of CA19-9 further support the interpretation that the hepatic lesions represented metastatic disease responsive to S-1.

An important limitation of the present case is the absence of histopathological confirmation of the hepatic lesions. Their metastatic nature was inferred from the presence of 6 lesions involving both hepatic lobes, including the largest lesion measuring 25 × 15 mm in the lateral segment, together with hypoenhancement on contrast-enhanced CT and diffusion abnormalities on MRI, in the setting of histologically confirmed pancreatic adenocarcinoma and markedly elevated CA19-9. The parallel regression of the pancreatic and hepatic lesions and normalization of CA19-9 further supported this clinical interpretation.

Recent evidence supports the value of advanced imaging for detecting and characterizing metastatic disease in pancreatic cancer. Molecular imaging may provide additional information regarding tumor biology and facilitate individualized assessment [10]. In a prospective pilot study of high-risk pancreatic ductal adenocarcinoma, diffusion-weighted MRI detected previously occult liver metastases in 34.2% of patients and demonstrated a sensitivity of 86.7% for hepatic metastases [11]. MRI characteristics of liver metastases, including those originating from pancreatic cancer, have also been shown to correlate with histopathological features [12]. Nevertheless, even highly suggestive imaging findings cannot substitute for histopathological confirmation. In the present case, liver biopsy was not performed because the pancreatic primary tumor had already been histologically confirmed, the multiple bilobar hepatic lesions were strongly suggestive of metastases, and additional biopsy was not expected to alter the treatment strategy. Alternative diagnoses therefore cannot be completely excluded, and we consistently describe the hepatic lesions as radiologically suspected rather than pathologically proven metastases.

S-1 consists of tegafur, a prodrug of 5-fluorouracil (5-FU), combined with gimeracil, which inhibits 5-FU degradation, and oteracil, which reduces gastrointestinal toxicity [13]. In the current treatment landscape, updated guidelines have incorporated NALIRIFOX among first-line treatment options for metastatic pancreatic cancer, alongside established combination regimens such as FOLFIRINOX and gemcitabine plus nab-paclitaxel [14]. These developments underscore that S-1 monotherapy is not a preferred regimen for all patients with metastatic disease and should be considered selectively according to patient factors such as age, performance status, tolerability, and treatment preference.

Another important limitation concerns the uncertain etiology of the terminal clinical deterioration. Approximately 49 months after initiation of S-1, the patient developed bilateral ground-glass and interstitial pulmonary opacities together with new-onset ascites. S-1-associated interstitial lung disease has previously been reported [15], and drug-induced interstitial lung disease related to S-1 was clinically suspected in the present case. However, the diagnosis was not pathologically confirmed. Although testing for coronavirus disease 2019 and influenza was negative and serological evaluation for autoimmune disease was unrevealing, pulmonary infection could not be completely excluded. No dedicated cardiac evaluation was performed. Furthermore, neither lung biopsy nor cytological examination of the ascites was performed. Therefore, pulmonary lymphangitic carcinomatosis and peritoneal progression also remained possible explanations.

Accordingly, the terminal event cannot be definitively attributed to either S-1-induced interstitial lung disease or malignant progression. We therefore report May 8, 2026, as the last date on which no definite radiological progression was documented, rather than assuming that the patient remained progression-free until death. This uncertainty does not negate the exceptionally durable response documented before the terminal event: the objective response was maintained for at least approximately 46 months, and CA19-9 remained within the normal range at the terminal admission.

The biological basis for such an exceptional response remains unknown. Individual differences in tumor biology and fluoropyrimidine sensitivity may have contributed to the prolonged response observed in this patient. Further molecular characterization of exceptional responders may help identify biomarkers associated with unusually prolonged benefit from fluoropyrimidine-based therapy. Molecular profiling of the tumor, including KRAS mutation status, microsatellite instability/mismatch repair status, and BRCA1/2 or other homologous recombination-related alterations, was not performed. Therefore, whether an unusual molecular background contributed to the exceptional treatment sensitivity cannot be determined in this patient. Although such molecular features could theoretically have influenced tumor biology and treatment response, any specific mechanism remains speculative in the absence of molecular testing.

## Conclusions

This case demonstrates an exceptionally durable response to first-line S-1 monotherapy in pancreatic adenocarcinoma with radiologically suspected synchronous multiple liver metastases. Although the hepatic lesions were not histopathologically confirmed, their radiological characteristics and clinical context were highly suggestive of metastatic disease. This case highlights the heterogeneity of treatment response in pancreatic cancer and suggests that selected patients may derive substantial benefit from S-1 monotherapy. Further studies are needed to identify the clinical and molecular characteristics associated with exceptional responses to S-1.

## References

[1] Conroy T, Desseigne F, Ychou M (2011). FOLFIRINOX versus gemcitabine for metastatic pancreatic cancer. N Engl J Med.

[2] Von Hoff DD, Ervin T, Arena FP (2013). Increased survival in pancreatic cancer with nab-paclitaxel plus gemcitabine. N Engl J Med.

[3] Ueno H, Ioka T, Ikeda M (2013). Randomized phase III study of gemcitabine plus S-1, S-1 alone, or gemcitabine alone in patients with locally advanced and metastatic pancreatic cancer in Japan and Taiwan: GEST study. J Clin Oncol.

[4] Mizuno N, Sawaki A, Yamao K (2006). Resolution of liver metastases in response to S-1 monotherapy in advanced pancreatic cancer--a case report [Article in Japanese]. Gan To Kagaku Ryoho.

[5] Yoshino T, Fukutomi A, Boku N (2006). Chemotherapy-naïve advanced pancreatic cancer with multiple liver metastases successfully treated by S-1 monotherapy--a case report [Article in Japanese]. Gan To Kagaku Ryoho.

[6] Sato R, Kawamura M, Kawamura T, Sasaki K, Ushigome T, Nyumura Y (2012). A case report of complete response to low-dose S-1 monotherapy for pancreatic cancer in elderly patient [Article in Japanese]. Gan To Kagaku Ryoho.

[7] Saso K, Murata M, Asai K (2015). A case of pancreatic cancer with multiple liver metastases that developed postoperatively and showed a complete response with S-1 monotherapy [Article in Japanese]. Gan To Kagaku Ryoho.

[8] Miyata T, Okamura Y, Sugiura T (2017). Pathological complete response after S-1 therapy for an unresectable pancreatic head cancer with liver metastasis [Article in Japanese]. Jpn J Gastroenterol Surg.

[9] Yamaga R, Tezuka K, Sugawara S, Watanabe T, Hirai I, Suzuki T, Kimura W (2018). S-1 induced discoid lupus erythematosus-like lesions and long-term complete response for para-aortic lymph node recurrence of pancreatic ductal adenocarcinoma: a case report. Surg Case Rep.

[10] Fathi M, Taher HJ, Al-Rubiae SJ (2024). Role of molecular imaging in prognosis, diagnosis, and treatment of gastrointestinal cancers: an update on new therapeutic methods. World J Methodol.

[11] Charbonneau J, Noël P, Ouellet JF (2025). Diagnostic value of diffusion-weighted MRI for the detection of peritoneal and liver metastases in high-risk pancreatic ductal adenocarcinoma: a prospective pilot study. Ann Surg Oncol.

[12] Barajas Ordonez F, Gottschling S, Eger KI, Borggrefe J, Jechorek D, Surov A (2024). MRI analysis of relative tumor enhancement in liver metastases and correlation with immunohistochemical features. Insights Imaging.

[13] Satoh T, Sakata Y (2012). S-1 for the treatment of gastrointestinal cancer. Expert Opin Pharmacother.

[14] Conroy T, Ducreux M (2025). ESMO Clinical Practice Guideline Express Update on the management of metastatic pancreatic cancer. ESMO Open.

[15] Yamane H, Kinugawa M, Umemura S (2011). An oral fluoropyrimidine agent S-1 induced interstitial lung disease: a case report. World J Clin Oncol.

